# Access to Primary Care Telemedicine and Visit Characterization in a Pediatric, Low-Income, Primarily Latino Population: Retrospective Study

**DOI:** 10.2196/57702

**Published:** 2024-12-17

**Authors:** Priya R Pathak, Melissa S Stockwell, Mariellen M Lane, Laura Robbins-Milne, Suzanne Friedman, Kalpana Pethe, Margaret C Krause, Karen Soren, Luz Adriana Matiz, Lauren B Solomon, Maria E Burke, Edith Bracho-Sanchez

**Affiliations:** 1 Division of Child and Adolescent Health Department of Pediatrics Vagelos College of Physicians and Surgeons, Columbia University New York, NY United States; 2 NewYork-Presbyterian Hospital New York, NY United States; 3 Department of Population and Family Health Mailman School of Public Health Columbia University New York, NY United States; 4 Division of Community and Population Health NewYork-Presbyterian Hospital New York, NY United States

**Keywords:** telemedicine, telehealth, pediatric primary care, COVID-19 pandemic, disparities, primary care, pediatrics, portals, access, accessibility, accessible, use, demographics, low income, Latino, Hispanic, Spanish, mobile phone

## Abstract

**Background:**

Since the COVID-19 pandemic, telemedicine has been widely integrated into primary care pediatrics. While initial studies showed some concern for disparities in telemedicine use, telemedicine uptake for pediatric patients in a low-income, primarily Latino community over a sustained period has yet to be described.

**Objective:**

We aimed to assess the relationship between demographics, patient portal activation, and telemedicine visits, as well as characterize diagnoses addressed in telemedicine, in a low-income, primarily Latino population over time.

**Methods:**

A multidisciplinary team conducted outreach for telemedicine and patient portal activation with the adoption of a new electronic health record. Data were collected on all in-person and telemedicine visits from February 2020 through April 2021 for 4 community-based pediatric practices. The outcomes included patient portal activation, telemedicine use, and reason for telemedicine visits. Bivariate tests and multivariate regression analyses were conducted to assess the independent effects of demographics on the likelihood of portal activation and having a telemedicine visit. Telemedicine diagnoses were categorized, and subanalyses were conducted to explore variations by age and month.

**Results:**

There were 12,377 unique patients and 7127 telemedicine visits. Latino patients made up 83.4% (n=8959) of the population. Nearly all patients (n=10,830, 87.5%) had an activated portal, and 33.8% (n=4169) had at least 1 telemedicine visit. Portal activation decreased with age >2 years (2-4 years: adjusted odds ratio [aOR] 0.62, 95% CI 0.51-0.76; 5-11 years: aOR 0.28, 95% CI 0.23-0.32; 12-14 years: aOR 0.29, 95% CI 0.23-0.35; and 15-17 years: aOR 0.46, 95% CI 0.36-0.58). Spanish-speaking (aOR 0.52, 95% CI 0.45-0.59) and non-Latino patients (aOR 0.64, 95% CI 0.54-0.76) had decreased odds of activation and having a telemedicine visit (aOR 0.81, 95% CI 0.74-0.89 and aOR 0.71, 95% CI 0.62-0.81, respectively). The top 5 diagnostic categories for telemedicine were infectious disease (n=1749, 26.1%), dermatology (n=1287, 19.5%), gastrointestinal (n=771, 11.7%), well and follow-up care (n=459, 7%), and other specialty-related care (n=415, 6.3%). Infectious disease showed the most variation over time. Age-based patterns included a decrease in the proportion of infectious disease diagnoses by increasing age group and a higher proportion of well and follow-up care in older ages. Additional telemedicine diagnoses included common infant concerns for patients younger than 2 years of age; pulmonary, asthma, and allergy concerns for toddler or school-age children; behavioral health concerns for younger adolescents; and genitourinary and gynecologic concerns for older adolescents.

**Conclusions:**

The high engagement across demographics suggests feasibility and interest in telemedicine in this low-income, primarily Latino population, which may be attributable to the strength of outreach. Language-based disparities were still present. Telemedicine was used for a wide range of diagnoses. As telemedicine remains a vital component of pediatric health care, targeted interventions may enhance engagement to serve diverse pediatric patient populations.

## Introduction

Before the COVID-19 pandemic, telemedicine was described as having the capacity to revolutionize pediatric care provision [1]. Despite this promise, only 15% of pediatricians reported using telemedicine in 2016 [2]. The most cited barriers then included insufficient payment, a lack of confidence in diagnoses that were made through telemedicine, patient reluctance and perceived lack of usefulness, and insufficient infrastructure [2,3]. The COVID-19 public health emergency triggered a rapid national shift to telemedicine, and decreased regulations and better payment parity allowed its adoption. As telemedicine has expanded, concerns about its potential to exacerbate health disparities have been raised [4-6]. The limited data that exist show that low digital health literacy, cultural preferences, and limited access to the internet and technological devices may limit engagement in telemedicine visits for certain populations [7]. Given the wide adoption of telemedicine, it is important to understand patterns of telemedicine uptake and use in diverse communities. In this study, we aim to characterize telemedicine visits for over a year during the pandemic in a low-income, primarily Latino population.

## Methods

### Study Environment

This study evaluated the patterns of outpatient telemedicine use among families of patients younger than 18 years of age from 4 community-based pediatric practices affiliated with NewYork-Presbyterian Hospital/Columbia University Irving Medical Center (NYP/CUIMC), New York. These practices are part of an ambulatory care network staffed by a single pediatric group practice using a common electronic health record (EHR) and have around 19,000 patients. The practices serve a primarily Latino and publicly insured population and provide primary care and refer out for subspecialty care. Demographic and visit data were collected for all patients with in-person or telemedicine visits during the study period. The study period spanned from February 2020 when a new EHR system was launched (described in the Telemedicine Implementation section), and data collection extended through April 2021.

### Telemedicine Implementation

In February 2020, a new EHR, Epic Systems, was launched across NYP/CUIMC. When the COVID-19 pandemic began in March 2020, NYP/CUIMC rapidly expanded telemedicine capacity across the network. Pediatric telemedicine was still in the pilot phase before the pandemic, orchestrated by 1 clinical champion. Early in the pandemic, there was an expansion of staff outreach. Extensive bilingual phone outreach for patient portal activation began in March 2020. Teams prioritized rapid patient enrollment due to the ongoing pandemic. Telemedicine capabilities were quickly expanded to accommodate acute care concerns and follow-up needs. Telemedicine visits were conducted through an Epic Systems video app. Patients were required to have access to a smartphone, tablet, or computer with internet capacity. If using a mobile device, they needed to download and install the Epic MyChart app, and then register for an account, which can be done in English or Spanish.

Although patients had the option to self-enroll digitally there was significant outreach to assist patients with the enrollment process. In March 2020, teams were created for the express purpose of patient enrollment. The bilingual team (Spanish or English) consisted of 17 staff and was interpreter supported for other languages. Administrators pulled data from the prior EHR and created detailed spreadsheets of all patients for enrollment. Staff was trained in enrollment, including health care proxy setup, as well as data management to track enrollment. There were rapid cycle performance improvement meetings to optimize protocols. Staff called patients and parents and assisted them in enrolling portal accounts. In some cases, families were assisted to create email accounts, as this was required for account access. A subset of patients with high risk was additionally prioritized by the primary care sites for outreach. Patients were deemed high risk by the criteria of the federal Maternal and Child Health Bureau’s children with special health care needs designation [8]. Many high-risk patients had a community health worker or care manager on their care team, and these staff also assisted in setting up the portal account. In some cases, community health workers used video calls to aid in setting up portal access. Additionally, if patients did not already have a portal account at the time of an in-person visit, providers or staff at the clinic could assist with portal access in person. When outreach was completed over the phone to set up portal access, the staff member confirmed portal setup by asking if the patients received an automated welcome message or if they could see their appointments or other portal components after setup. In our EHR, each patient chart clearly indicates whether they have activated their portal, and this is also confirmed by staff and providers at patient appointments.

Most telemedicine appointments were initiated when parents requested an appointment for an acute concern. The nursing staff triaged the concern and determined whether to schedule an in-person or telemedicine visit. Triaging protocols were agreed upon in advance by nursing and physician representatives and evolved throughout the COVID-19 pandemic. For patients with a primary language other than English, team members used a third‐party interpreter service.

### Study Variables and Outcomes

Patient demographics including age, sex, race, ethnicity, preferred language, and insurance status were extracted from the EHR for all individual patients who were seen in person or on telemedicine. All ages reported refer to the patient’s age. Race and ethnicity data were collected by self-report.

Our institution has been part of a multi-institutional framework to more accurately collect race and ethnicity data in service of identifying and addressing health disparities, which has now been adapted by many other large health systems across the country since 2020 [9,10]. We collect information from patients using the standard US Office of Management and Budget 2-question format for race and ethnicity as well as 2 follow-up questions specified by the New York State Department of Health regarding granular ethnicity and granular race. Race and ethnicity values are typically captured during registration, either by front desk staff or on a tablet used by patients to electronically register at appointments. Patients were informed that it is optional to self-report and that patient information is confidential.

Data were collected for all patient visits within the study period, including both in-person and telemedicine visits. The primary study outcomes were patient portal activation (needed for telemedicine visits) and the presence of a video visit, referred to herein as a telemedicine visit. Another variable characterized was the primary diagnosis from telemedicine visits.

For diagnosis data, categories were created by the research team (MSS, PRP, and EB-S) after a review of the primary diagnosis of visits in our initial data in a qualitative manner. Two team members (MSS and EB-S) reviewed and classified all diagnoses manually into categories. A third team member (PRP) then reviewed all of the diagnosis classifications, grouped categories together with iterative feedback from authors, and clarified any ambiguous diagnoses. Each visit diagnosis as coded in the EHR was only assigned to 1 particular category. When there was ambiguity regarding categorization, the full-visit documentation was reviewed to most accurately categorize the diagnosis, and each of these decisions was reviewed by 2 authors (PRP and EB-S). The categorization was iterative with continuous refinement and validation until a consensus was reached.

### Statistical Analysis

Analyses on demographic data and the presence of portal activation reflect the dataset of individual patients (n=12,377). Descriptive outcomes of diagnoses seen on telemedicine reflect unique telemedicine visits (n=7127), in which patients who have had multiple telemedicine visits are represented more than once. Chi-square analyses were conducted to examine associations between patient demographics and portal activation and having a telemedicine visit (separately). Multivariable logistical regression analyses were then conducted. Any variable that had a significant univariate 2-tailed *t* test at *P*≤.10 was used as a variable for the multivariable analysis, based on the literature on purposeful selection [11,12]. For portal activation, covariates included were age (categorical: <2, 2-4, 4-11, and 12-18 years), language (categorical: Spanish, English, and other), and ethnicity (Latino and non-Latino). For the presence of a telemedicine visit, covariates included were age (categorical: <2, 2-4, 4-11, and 12-18 years), language (categorical: Spanish, English, and other), ethnicity (Latino and non-Latino), and race (White, Black, and other). There were no additional covariates in the model.

On the telemedicine visit data (n=7127), diagnoses from telemedicine visits were categorized and described overall. Subanalyses were conducted to explore variations in diagnoses by age and month. Statistical analyses were performed using SPSS software (version 28; IBM Corp). The significance level was set at α=.05.

### Ethical Considerations

The study protocol was reviewed and approved by the Columbia University Irving Medical Center Institutional Review Board (#AAAS8260). This institutional review board approval covered secondary analysis without additional consent. Data collection, storage, analysis, and reporting adhered to institutional guidelines. There was no compensation of participants, as only secondary data were used.

## Results

Overall, there were 12,377 unique patients with visits during the time period. Half (n=6183, 50%) of the patients were female, 52.6% (n=6509) were younger than 5 years of age, 44.6% (n=5497) were Spanish speaking, 83.4% (n=8959) were Latino, and 21% (n=2160) were Black (Table 1). Insurance data from televisits showed that 95% (n=6360/6695) of patients were publicly insured. Of all patients (N=12,377), 10,830 (87.5%) had activated the patient portal. Of all patients, 33.8% (n=4169) had at least 1 telemedicine visit. Of those who activated their portal account and subsequently had an in-person or video visit, 38.5% (n=4169/10830) had a video visit. Of those with a telemedicine visit, 37.7% (n=1572/4169) had more than 1 telemedicine visit, the median number of visits was 1 (IQR 1-2; range 1-15).

The highest proportion of patient portal activation was for patients at the 2 extremes of age ranges. Of all patients younger than 2 years of age (n=4390), 94.1% (n=4129) had activated the portal, and of those between 15 and 17 years of age (n=1090), 87.2% (n=950) had portal activation. School-age children (5-11 years; n=3503) had the lowest rate at 80.1% (n=2807). On multivariable logistic regression analysis, decreased odds of portal activation were seen with age >2 years (2-4 years: adjusted odds ratio [aOR] 0.62, 95% CI 0.51-0.76; 5-11 years: aOR 0.28, 95% CI 0.23-0.32; 12-14 years: aOR 0.29, 95% CI 0.23-0.35; and 15-17 years: aOR 0.46, 95% CI 0.36-0.58). Decreased odds of portal access were seen for those speaking Spanish (aOR 0.28, 95% CI 0.23-0.32) or another non-English non-Spanish language (aOR 0.62, 95% CI 0.47-0.81) and being non-Latino (aOR 0.64, 95% CI 0.54-0.76; Table 1). Demographic characteristics associated with having had at least 1 telemedicine visit were similar but not the same as those for portal activation. School-age children were again least likely to have had at least 1 visit compared to those younger than 2 years of age (aOR 0.80, 95% CI 0.72-0.90), but adolescents had slightly greater odds of having a visit (aOR 1.17, 95% CI 1.00-1.36). Decreased odds of a visit were seen for those who spoke Spanish (aOR 0.81, 95% CI 0.74-0.89) or other non-English and non-Spanish language (aOR 0.65, 95% CI 0.52-0.81) or were non-Latino (aOR 0.71, 95% CI 0.62-0.81). Those who did not identify as Black or White also had decreased odds of having a telemedicine visit (aOR 0.89, 95% CI 0.81-0.98; Table 1).

**Table 1:** Patient portal activation and the presence of telemedicine visits for unique patients by demographics.

		All participants (N=12,377), n (%)	Portal activation, n (%)^a^	Portal nonactivation, n (%)^a^	*P* value		aOR^b^ (95% CI)^c^		Telemedicine visit, n (%)^a^		No telemedicine visit, n (%)^a^		*P* value		aOR (95% CI)^d^	
**Age range (years)**						*<.001^e^*								*<.001*		
	<2	4390 (35.5)	4129 (94.1)	261 (5.9)			Reference		1556 (35.4)		2834 (64.6)				Reference	
	2-4	2119 (17.1)	1908 (90)	211 (10)			*0.62 (0.51-0.76)*		711 (33.6)		1408 (9.4)				0.94 (0.83-1.06)	
	5-11	3503 (28.3)	2807 (80.1)	696 (19.9)			*0.28 (0.23-0.32)*		1060 (30.3)		2443 (69.7)				*0.80 (0.72-0.90)*	
	12-14	1275 (10.3)	1034 (81.1)	241 (18.9)			*0.29 (0.23-0.35)*		426 (33.4)		849 (66.6)				0.94 (0.81-1.09)	
	15-17	1090 (8.8)	950 (87.2)	140 (12.8)			*0.46 (0.36-0.58)*		434 (39.8)		656 (60.2)				*1.17 (1.00-1.36)*	
**Language**						*<.001*								*<.001*		
	English	6212 (50.3)	5607 (90.3)	605 (9.7)			Reference		2221 (35.8)		3991 (64.2)				Reference	
	Spanish	5497 (44.6)	4655 (84.7)	842 (15.3)			*0.52 (0.45-0.59)*		1781 (32.4)		3716 (67.6)				*0.81 (0.74-0.89)*	
	Other	629 (5.1)	534 (84.9)	95 (15.1)			*0.62 (0.47-0.81)*		175 (27.8)		454 (72.2)				*0.65 (0.52-0.81)*	
**Sex**						.33								.62		
	Male	6192 (50)	5399 (87.2)	733 (12.8)			—^f^		2107 (34)		4085 (66)				—	
	Female	6183 (50)	5427 (87.8)	756 (12.2)			—		2078 (33.6)		4105 (66.4)				—	
**Ethnicity**						*.04*								*<.001*		
	Latino	8959 (83.4)	7889 (88.1)	1070 (11.9)			Reference		3188 (35.6)		5771 (64.4)				Reference	
	Non-Latino	1787 (16.4)	1543 (86.3)	244 (13.7)			*0.64 (0.54-0.76)*		529 (29.6)		1258 (70.4)				*0.71 (0.62-0.81)*	
**Race**						.37								*.04*		
	Black	2160 (21)	1904 (88.1)	256 (11.9)			—		710 (32.9)		1450 (67.2)				0.95 (0.82-1.08)	
	White	3164 (30.8)	2778 (87.8)	386 (12.2)			—		1146 (36.2)		2018 (63.8)				Reference	
	Other	4949 (48.2)	4308 (87)	641 (13)			—		1709 (34.5)		3240 (65.5)				*0.89 (0.81-0.98)*	

^a^Percentages use the n value in the “All participants” column as the denominator.

^b^aOR: adjusted odds ratio.

^c^Covariates for portal activation are age, language, and ethnicity.

^d^Covariates for telemedicine visits are race, age, language, and ethnicity.

^e^Values in italics format indicate statistically significant findings.

^f^Not applicable.

**Figure 1:**
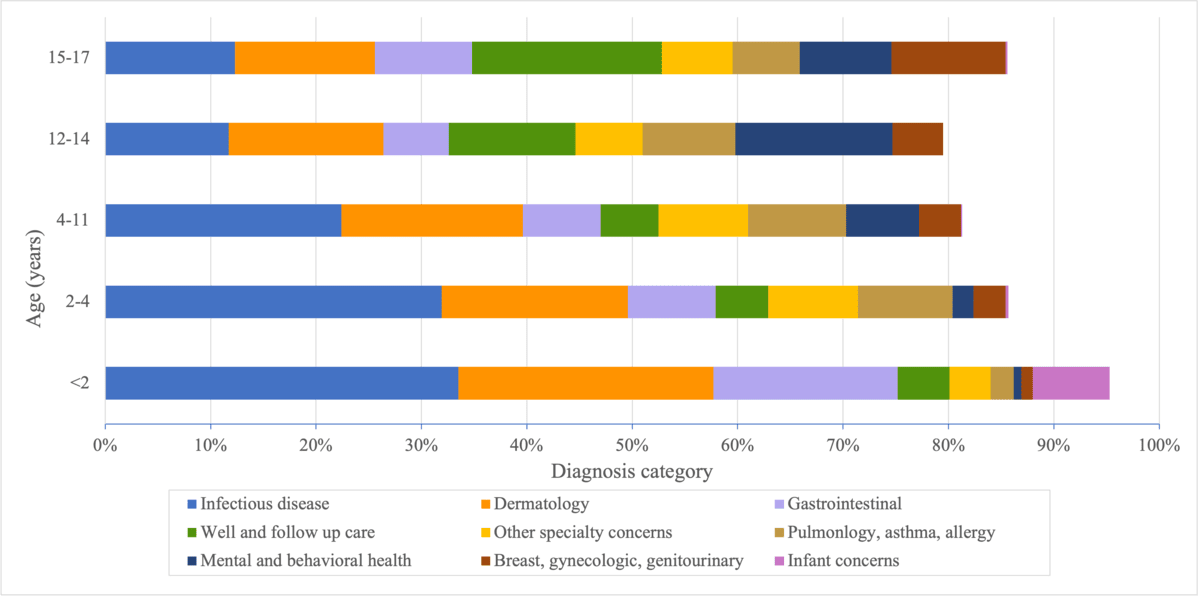
Age-based variation in telemedicine diagnosis categories.

During the study period, there were 7127 telemedicine visits. The top 5 diagnoses categories were infectious (n=1860, 26.1%), dermatology (n=1389, 19.5%), gastrointestinal (n=771, 11.7%), well and follow-up care (n=499, 7%), and other specialty-related care (n=449, 6.3%). Patterns in telemedicine diagnoses differed by age (Figure 1). Infectious disease, the most common overall diagnosis category, was high in all age groups. However, there was a steady decrease in the proportion of the infectious disease category by increasing age group. For example, in visits for children of 0-2 years of age (n=2663), infectious disease made up 33.5% (n=892) of all visits versus only 12.3% (75/610) of visits for adolescents of 15-17 years of age. Dermatologic concerns were the second-most predominant diagnosis category for all age groups. There were also age-based patterns in well and follow-up care, as well care was more predominant for older age groups. For the 0- to 2-year age group (n=4390), there was a high proportion of gastrointestinal concerns (n=467, 17.5%) as well as other infant concerns (n=195, 7.3%), which included breastfeeding problems, colic, nasolacrimal duct stenosis, and teething. In the 2- to 4-year age group (and 5- to 11-year age group), pulmonary, asthma, and allergy-related diagnoses were the third-most prevalent concern. In the 12- to 14-year age group, the most predominant category was behavioral and mental health concerns (n=93). For the 15- to 17-year age group, genitourinary, gynecologic, and breast concerns were within the top 5 categories (n=66), which was not seen for any other age group.

**Figure 2:**
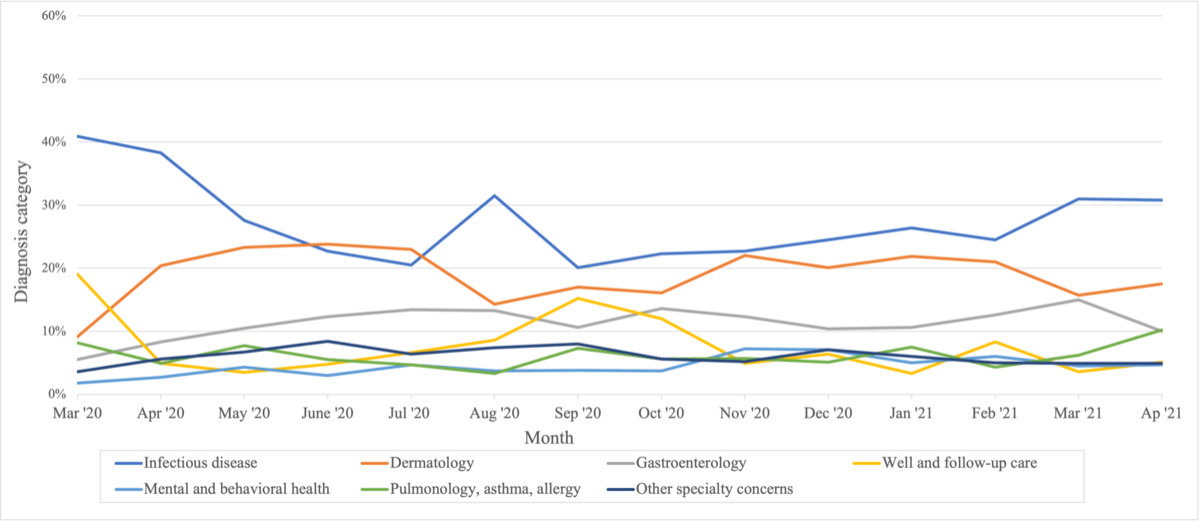
Temporal variation in telemedicine diagnosis categories.

Patterns in diagnoses also changed by month (Figure 2). The number of video visits overall rose in spring 2020, peaking in May 2020 (n=629) and then decreasing in June (n=609), July (n=536), and August (n=520). After a small bump in September (n=558), telemedicine visits made up between 400 and 530 visits per month. At the onset of telemedicine visits in March 2020, the infectious disease made up the highest proportion of visits (45/110, 40.9%), and well and follow-up care accounted for an additional 19.1% (21/110). The proportion of infectious disease cases declined in the spring, then rose again to peak by August 2020, and after this, consistently made up the highest proportion of telemedicine diagnoses. In our clinic system, as in-person visits became more available later in the pandemic, infectious disease diagnoses continued to make up the highest proportion of telemedicine visits. After the initial high proportion of well and follow-up care visits, these visits declined rapidly in April 2020, then rose again to a peak in August and September 2020. In the spring of 2020, the proportion of dermatology diagnoses as a fraction of all diagnoses rose steadily until June 2020 when dermatology diagnoses made up of 23.8% (145/608 visits in June) of televisits. After this, dermatologic diagnoses made up between one-tenth and one-quarter of all cases. Other diagnosis categories showed less variation over time. After a slow increase over the spring of 2020, gastrointestinal diagnoses made up the third-most common category for most months. Mental and behavioral health made up a very small proportion of cases initially (2/110, 1.8%) but slowly rose up to 7.2% (33/461) by December 2020, after which it made up between 4.7% and 7.1% (from 23/491 to 32/453) of cases.

## Discussion

### Principal Findings

In this study, which assessed telemedicine use immediately after rollout in the early pandemic period, we found that widespread access to telemedicine, as measured by portal activation, is possible in a low-income, primarily Latino community. In this population, telemedicine appointments were used for a wide array of diagnoses across the age spectrum. Our findings have implications for the use of telemedicine to support and augment pediatric primary care provision for diverse patient populations.

### Comparison to Prior Work

In our study population, which was largely publicly insured, we found that of all patients seen, 87.5% (10,830/12,377) had an active portal account, and a third had at least 1 telemedicine visit with the use of multipronged bilingual outreach for portal activation. Schenker et al [13] studied a pediatric primary care practice during the first 5 months of the pandemic and found that public insurance was a significant negative predictor of having had a video visit. We did identify some demographic disparities, particularly in language. Non-English speakers had decreased odds of portal activation and of having a video visit, with the lowest visits among those who spoke neither English nor Spanish. Similar to our findings, Blundell et al [14] found that in a pediatric dermatology practice, patients speaking Spanish had lower rates of having an email address in the EHR and lower rates of having an activated patient portal account. Conversely, Schenker et al [13] did not find language to be a significant predictor of telemedicine use. Rodriguez et al [15] found that patients with limited English proficiency had less access to telemedicine and also reported worse experiences with video visits as compared to in-person visits. The authors posit that this may be related to difficulty with interpreter use, providers or patients perceiving video visits to be less effective, or digital barriers. It is likely that these barriers are also present in our patient population [15]. While our clinical sites are fully bilingual and have a strict interpreter policy with access to all languages, outreach in different languages to let families know about multilingual telehealth could be helpful both in person at visits by front desk staff and providers and by nurses when providing triage. Paper sheets that have portal access codes and set-up instructions could be made available in multiple languages (not only English or Spanish) for staff to distribute. Other interventions in the literature include the use of patient navigators for telemedicine [16]. The use of telemedicine navigators was found to benefit physicians and patients along with being cost-effective. Some authors have also described digital rooming [17]. This involves the use of medical assistants to call patients prior to telemedicine appointments for help with connecting to the visit digitally and found some success with increasing the likelihood of patients appropriately connecting to the visit. This is not specifically targeted at non-English and non-Spanish speakers; however, if adapted to serve these populations, for example, with the routine use of interpreters, it could also function to increase accessibility. In our literature review, we find that both interventions have been used to a greater extent for adult populations [16,17].

Interestingly, in our study population, being Latino was associated with higher odds of both portal activation and having at least 1 visit. It is important to note that we consider our race and ethnicity data to reflect social constructs, and differences in outcomes by race and ethnicity provide an understanding of structural health care disparities that may further drive inequities [18]. Understanding differences in telemedicine use by race and ethnicity provides evidence for targeted interventions to decrease institutional or systemic bias and racism. Looking across the pediatric and adult literature, the relationship of telemedicine use by race and ethnicity has been mixed. Several studies have described that White patients are more likely to use telemedicine [13,19]. Some studies early in the pandemic show decreased telemedicine use in Latino or Hispanic populations compared to White patients [20,21]. However, others such as Samson et al [22] found that Hispanic and Asian patients had higher use of telemedicine than White or Black patients. Some authors have hypothesized that there are modifying factors, such as geographical differences, or community preferences that affect use within ethnic groups [23].

### Strengths and Limitations

It is not entirely clear why our Latino patient use was higher than non-Latino use. We believe that the differences seen in our study are at least partially attributable to our outreach. Our team aimed to increase access for all patients and also focused on decreasing barriers for Latino and Spanish-speaking patients. A multidisciplinary approach to enrollment was critical—for example, staff, patient financial advisors, care managers, community health workers, and care providers could all assist with enrollment and interact with patients in different contexts. Our enrollment occurred with major tenets of cultural competency in place, described as the “tailoring of health care delivery to meet patients’ social, cultural and linguistic needs” [24]. Many staff on the enrollment team, particularly staff, patient financial advisors, and community health workers, come from the same community and regional background as our patients. Many of them identify as Latino and spoke Spanish. Anecdotally, staff reported that when they were able to identify with families, they could provide more trust and reassurance for families who had reservations about the process of enrollment or telemedicine participation. Further, as part of the drive for patient enrollment, staff were supported to iterate enrollment protocols, particularly to target barriers for families with respect to language, culture, and health literacy such as using language for enrollment that was patient-responsive and reflective of the local Spanish dialect. We believe that devoting time and resources to patient enrollment and engaging patient-facing staff to increase our cultural competency are reflected in our results of Latino enrollment.

In regard to the use of video visits, there may be other systematic differences between non-Latino and Latino populations that make telemedicine visits more convenient or accessible for Latino patients in our study population as compared to in-person visits. These could be social determinants of health, such as job or transportation-related factors, or even demographic factors such as parent age or parental comfort with technology used for video visits. Our high level of engagement across demographics, including in Latino patients, suggests feasibility and interest in telemedicine in this low-income, diverse population and underscores the importance of outreach.

Telemedicine engagement, as indicated both by portal activation and telemedicine visits, also showed age-related variation. Patients younger than 2 years of age and those between 15 and 17 years showed the most engagement. Other authors have reported similar patterns. Walters et al [19] reported families of patients younger the 1 year of age as more likely to use telemedicine, whereas Schenker et al [13] found that older patients were more likely to have had video visits. The frequency of well-child visits for infants and a higher rate of contact with the health care system may have influenced higher portal activation rates for children younger than 2 years of age. In our health care system, adolescents are given individual portals with enhanced confidentiality at 12 years of age. Older teens in the 15- to 17-year age group likely have the skills to independently access their portal and obtain telemedicine and may find televisits a convenient way to access care. Research on adolescents and caregivers has shown that telemedicine is widely acceptable for this age group [25,26]. Of all age groups, school age children (5-11 years) had the lowest proportion of telemedicine use. Possible barriers may be that parents of elementary school children have a stronger preference for in-person visits or the times telehealth visits are offered are not amenable to parent or child schedules as compared to younger and older age groups.

We found that video visits were used for a wide range of diagnoses including acute care, well care, and mental health concerns. Video visit diagnosis patterns can be assumed to be guided by the needs of patients and families, as well as the triage protocols used by nursing staff who directed patients to in-person or video visits. Temporal variation in diagnoses illustrates the evolving nature of the COVID-19 pandemic, with a high prevalence of infectious disease case concerns in March 2020 and subsequent intermittent peaks. Well and follow-up care also peaked in the spring of 2020 when in-person care was reserved for more acute concerns but subsequently did not remain as common a reason for telemedicine. Overall, the categories of infectious disease, gastroenterology, and dermatology stayed high throughout the study period, showing that telemedicine may be a useful modality to address these common pediatric complaints. The persistence of the infectious disease category highlights the relevance of telemedicine to manage routine infectious conditions beyond COVID-19. In a qualitative study on telemedicine for acute respiratory concerns, parents reported that they prefer telemedicine because it is easier and it decreases wait time, disruptions in schedules, and exposure to other ill children in a waiting room [27]. Telemedicine may also help with use patterns elsewhere in a health care system. For example, Walters et al [19] asked caregivers who used telemedicine for acute concerns about potential alternatives and found that a substantial portion would have gone to the emergency department or urgent care center (16.5%) or opted not to seek care (11.3%) if telemedicine was not an option. Indeed, our most common categories—infectious disease, dermatologic, and gastrointestinal concerns—make a large component of pediatric urgent care and emergency department visits [28]. Our findings have potential implications for strengthening telemedicine policies, for example, improving triage algorithms for common diagnostic categories, so that telehealth visits provided by the medical home can serve as an effective alternative to using emergency services or meet the needs of patients who have barriers to in-person visits.

There were also notable patterns in diagnosis categories by age group. Though common in all age groups, infectious disease visits were less common for older individuals. Infectious disease was the most common diagnosis category for infants through school-aged children, but for younger adolescents, mental health was most predominant, and for older adolescents, it was well and follow-up care. Dermatologic concerns consistently ranked as the second-most predominant category for all groups. Additional associations by age included common infant concerns for patients younger than 2 years of age; pulmonary, asthma, and allergy-related concerns for school-age children; and genitourinary and gynecologic concerns for older adolescents. These age-dependent shifts in diagnoses emphasize the evolving health care needs of pediatric patients and the acceptability by both parents and providers to address a variety of concerns through telemedicine. Other studies have found age differences in telemedicine use [13,29] but have not examined age-related variation in diagnoses to the extent of this study. In the postpandemic era, both primary care pediatricians and caregivers expect to use telemedicine as a routine part of care provision for acute as well as chronic concerns [19,30-32]. As telemedicine further integrates into primary care practices, understanding age-based variation in use can help strengthen practice-level policies to appropriately and optimally meet the evolving needs of patients and caregivers.

This study has several limitations. First, our findings have geographical and temporal limitations for generalizability. Given that we studied telemedicine use with the onset of the pandemic from February 2020 to April 2021, we do not have a prepandemic comparison group. Our data here also reflect the unique time period in the first year of the pandemic when there was a relative lack of familiarity with web-based medicine. We know telehealth use has evolved during and beyond the pandemic. For example, Solo-Josephson et al [33] found that later in the pandemic, patients using telemedicine had a larger range of preferred languages spoken. The unique manner in which the COVID-19 pandemic progressed in New York City and the local health care and public policy responses are not necessarily representative of other geographical locations. Furthermore, given our community, our findings may be more generalizable to other populations with a predominant proportion of Latino patients or another similar ethnic majority. Other studies have described a similar pivot to telemedicine in pediatric care with the onset of the pandemic [13,14,27,30-32,34,35]. As compared to other institutions, the deployment of the Epic Systems EHR was a unique circumstance at the beginning of our project. Though portal activation was not required of all patients, there was a great deal of outreach to promote activation during the initial few months. The fact that we were able to capture activation in real time was significant, and our results indicate the importance of outreach with such new interventions. However, it is possible that telemedicine use would be different if there had not been a temporal overlap with the deployment of a new EHR. In addition, in our study, the outcomes measured were patient portal activation and the presence of a telemedicine visit. While these are important metrics, we do not describe other factors related to use, such as the reasons for choosing telemedicine over in-person visits, barriers to telemedicine, and patient satisfaction with telemedicine. Further, there were some limitations related to data categorization. Regarding demographic data, we recognize that EHRs have historically had inaccuracies in race and ethnicity data, particularly for Latino, Asian, Native American, and Pacific Islander patients [36]. As it relates to pediatric patients, EHRs and databases may also show discordance for children versus adults, given the additional challenges of gathering data for pediatric patients [10]. Our institution has been part of a multi-institutional framework, starting in 2020, to address some of these challenges. This campaign, We Ask Because We Care, is an exemplar of improvement in data collection. This methodology has now been adapted by other large health systems in the United States since 2020 [9,10]. The framework includes continuous measurement and monitoring with real-time dashboards. Finally, there were also some challenges related to the classification of the diagnosis data. In the diagnosis data, there were some diagnoses that could arguably fit into multiple categories (eg, chest pain as “cardiac” or “musculoskeletal”). We aimed to enhance validity and limit misclassification through detailed chart review where there was ambiguity, as well as iterative categorization and verification by multiple authors.

### Future Directions

Avenues for future research include longer-term analysis and qualitative exploration of barriers and facilitators of telemedicine in specific populations, including Latino and Spanish-speaking families, such as factors related to demographic or socioeconomic conditions. Another avenue of further study would be to investigate variations in preferences or satisfaction with telemedicine in different age groups, particularly in the postpandemic period.

### Conclusions

Our study provides valuable insights into the implementation of telemedicine in pediatric primary care, particularly for Latino patients, as well as use for common pediatric diagnoses across age groups and over time. As telemedicine has emerged as a vital component of pediatric health care, these findings can inform targeted interventions to enhance accessibility, improve engagement, and tailor telemedicine services to the diverse needs of pediatric patients and families.

## Abbreviations

aOR: adjusted odds ratio
EHR: electronic health record
NYP/CUIMC: NewYork-Presbyterian Hospital/Columbia University Irving Medical Center
